# Effects of neuroactive agents on axonal growth and pathfinding of retinal ganglion cells generated from human stem cells

**DOI:** 10.1038/s41598-017-16727-1

**Published:** 2017-12-01

**Authors:** Tadashi Yokoi, Taku Tanaka, Emiko Matsuzaka, Fuminobu Tamalu, Shu-Ichi Watanabe, Sachiko Nishina, Noriyuki Azuma

**Affiliations:** 1 Department of Ophthalmology and Laboratory for Visual Science, National Centre for Child Health and Development, Tokyo, Japan; 2 Department of Physiology, Faculty of Medicine, Saitama Medical University, Saitama, Japan

## Abstract

We recently established a novel method for generating functional human retinal ganglion cells (RGCs) from human induced pluripotent cells (hiPSCs). Here, we confirmed that RGCs can also be generated from human embryonic stem cells (hESCs). We investigated the usefulness of human RGCs with long axons for assessing the effects of chemical agents, such as the neurotrophic factor, nerve growth factor (NGF), and the chemorepellent factors, semaphorin 3 A (SEMA3A) and SLIT1. The effects of direct and local administration of each agent on axonal projection were evaluated by immunohistochemistry, real-time polymerase chain reaction (PCR), and real-time imaging, in which the filopodia of the growth cone served as an excellent marker. A locally sustained agent system showed that the axons elongate towards NGF, but were repelled by SEMA3A and SLIT1. Focally transplanted beads that released SLIT1 bent the pathfinding of axons, imitating normal retinal development. Our innovative system for assessing the effects of chemical compounds using human RGCs may facilitate development of novel drugs for the examination, prophylaxis, and treatment of diseases. It may also be useful for observing the physiology of the optic nerve *in vitro*, which might lead to significant progress in the science of human RGCs.

## Introduction

Optic nerve diseases, including congenital anomalies^1,2^, neuritis^3^, and glaucoma^4^, are the principal causes of severe visual impairment in humans. Despite the varied pathogenesis of optic nerve diseases, including genetic abnormalities^5,6^, inflammation, trauma^7^, demyelination^3^, and glaucoma, retinal ganglion cell (RGC) injury, resultant cell death occurs in all these diseases^8^. At the early stage of most of these diseases, the axon of the RGC, rather than the cell body, is the first part of the cell to be injured, and this induces cell body apoptosis^9,10^. Therefore, a better understanding of RGC axons is the key to elucidating the pathogenesis and the pathological conditions underlying these optic nerve diseases. In addition, the main physiological role of RGCs is to transmit retinal information to the brain, and to this end, RGCs have developed unusually long axons^11^. Therefore, in order to elucidate the pathogenesis of optic nerve diseases and the physiological functions of RGCs, as well as to treat optic nerve diseases, it is important that research extends beyond the current studies of animal models that employ axonal crush^12,13^ to also focus on *in vitro* generation of RGCs with characteristically long axons.

Numerous animal models of optic nerve diseases have been established for *in vivo* studies, including high ocular pressure models of mice, rats, sheep, and pigs^14–16^, optic nerve crush models, and glutamate/aspartate transporter-deficient mice for the assessment of normal-tension glaucoma^17^. These animal models provide useful tools for investigating pathogenesis and the resulting pathological condition of human diseases. However, possible differences between animal and human cells pose serious obstacles to the interpretation of the results in a clinical context, particularly in term of the evaluation of pharmacokinetics and drug effects. Moreover, in those *in vivo* models, the pathological status and drug effects are assessed mainly by evaluating the structure of the inner retina or the optic nerve by histopathology and optical coherence tomography^18^, as real-time observation of RGCs and their axons has been difficult. Thus, an *in vitro* system has a clear advantage in terms of allowing a more detailed, precise, direct, and real-time observation of RGCs.

Several *in vitro* models have been suggested as the *in vitro* glaucoma model, in which the effect of hydrostatic pressure on RGCs isolated from the retina of mice and other rodents were analysed^19^. However, these isolated cells were only sustainable on culture dishes for a few weeks, and did not exhibit the characteristic axons^20^. Therefore, these cells are considered unsuitable for use as *in vitro* glaucoma models, because they may not have sufficient resilience to tolerate stress, including hydrostatic pressure^19,21^ and N-methyl-D-aspartate (NMDA) supplementation^22^. Sustainable human RGCs with their characteristically long axons would be the most suitable models for discovering candidate drugs, but the cells are available exclusively from the retina of human cadavers using immunopanning^23^ and the number of available human cells is limited. Moreover, the characteristics and functions of the axons of RGCs, which play a crucial role in mechanisms involving optic neuropathies, have not been demonstrated in RGCs isolated from human cadavers. Consequently, establishment of sustainable RGCs with axons from human stem cells is highly desired.

We have recently established a novel method for generating RGCs with functioning and stable long axons from human induced pluripotent cells (hiPSCs). Our RGCs survive for approximately 50 days and are generated with an efficiency of 90%^24^. In this experiment, we employed 3-dimensional (3D) and 2-dimensional (2D) culture methods to culture floating aggregates of hiPSCs as embryoid bodies (EBs). EBs were induced into a retinal cell lineage and subsequently into 3D cultures of optic vesicles (OVs). OVs are the primordia of the retina and extrude from the EBs. EBs were then transferred to culture dishes as 2D cultures, which resulted in both the generation of RGCs at the marginal portion of the attached OVs and in the extension of long radial axons with physiological functioning.

Upon injury to the optic nerve, RGCs experience cell death, which is a physiologically irreversible phenomenon. Therefore, treatment of optic nerve diseases relies upon novel drug development for neuroprotection and nerve regeneration, and possibly upon the transplantation of human RGCs. In the current study, we show that RGCs can also be generated from human embryonic stem cells (hESCs) using the protocol we previously established for generating RGCs from hiPSCs^24^. We also investigated the utility of our RGCs, which contained numerous firm, long axons elongating in the same direction, for assessing the effects of administered neurotrophic and chemorepellent agents. We evaluated nerve growth factor (NGF) as a neurotrophic factor, and semaphorin 3A (SEMA3A) and SLIT1 as chemorepellent factors, in which various *in vitro* analyses of the nerve elongation pattern, nerve pathfinding, including real-time nerve pathfinding, and the growth cone, were carried out.

## Methods

### Culture of human embryonic stem cells and induced pluripotent stem cells

Human embryonic stem cells (hESCs) (HES0001_KhES-1) and induced pluripotent stem cells (iPSCs) (HPS0007_409B2, cell passage 29) were obtained from RIKEN BRC (Japan) and cultured as described previously^24^. Briefly, cells were maintained on mouse embryonic fibroblasts (MEFs) in primate ES medium (ReproCELL) supplemented with recombinant human basic fibroblast growth factor (Invitrogen). For passaging, cell colonies were detached using dissociation solution (ReproCELL) and detached clumps of hESCs and hiPSCs were broken into smaller portions. Cells were passaged at split ratios of 1:3 to 1:4.

The use of hESCs and hiPSCs in this study was approved by the Ethics Committee of the National Institute for Child and Health Development (NCCHD), Tokyo (approved #ES-Rin 261).

### Induction of differentiation in retinal ganglion cells

RGCs were induced from hESCs and hiPSCs based on methods that involve a serum-free floating culture of EB aggregates^25^ and our previously established approach^24^. For a short period, hESCs and hiPSCs were dissociated into single cells and were resuspended in retinal differentiation medium (RDM; G-MEM supplemented with 20% KSR, 0.1 mM nonessential amino acids, 1 mM pyruvate, 0.1 mM 2-mercaptoethanol, 100 U/mL penicillin, and 100 mg/mL streptomycin). After separation from the feeder cells, floating hESCs and hiPSCs were collected and seeded into V-bottomed plates (Sumitomo Bakelite, Tokyo, Japan) at 9,000 cells per well. On the second day (D2) after initiating suspension (D0), Matrigel (BD Bioscience) was added, and on D12, the aggregates were transferred to 24-well plates (BD Bioscience) and the medium was replaced with RDM containing 1.0% Matrigel and 1.0% fetal bovine serum (FBS). On D15, CHIR99021 (3 µM; Wako Pure Chemicals) and SAG (100 nM; Enzo Life Science) were added to the medium and the suspension culture was continued for another 3 days. EBs were transferred to retinal maturation medium (RMM; DMEM/F12-Glutamax medium containing the N2 supplement, 100 U/mL penicillin, and 100 mg/mL streptomycin) on D18 and cultured in the absence of FBS for 6 days. On D24, OVs started to extrude, and 1.0% FBS and retinoic acid (0.5 µM; Sigma−Aldrich) was added to the RMM. On D27, adhesion culture was initiated when EBs were transferred to poly-d-lysine/laminin-coated 24-well plates with the supplementation of brain-derived neurotrophic factor (100 ng/mL BDNF, R&D Systems) instead of retinoic acid.

### Real-time polymerase chain reaction

Real-time polymerase chain reaction (PCR) was performed as previously described^24^ using an RNeasy Mini Kit and analysed using a One Step SYBR PrimeScript PLUS real-time PCR Kit (Takara Bio Inc.) and StepONE Sequence Detection System (Applied Biosystems). The primers used in this study are listed in Supplementary Table S1.

**Figure 1:**
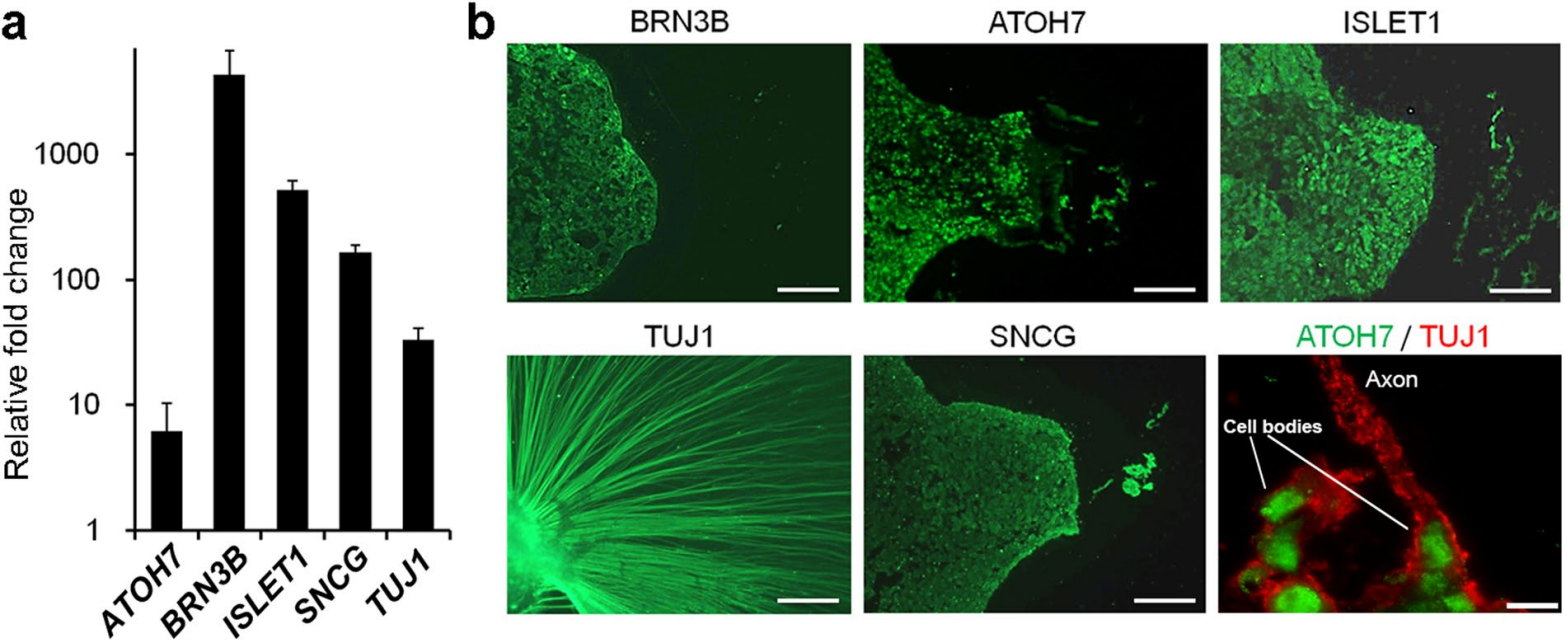
Identification of hESC-derived RGC markers. (**a**) Expression of representative RGC markers, *ATHO7, BRN3B, TUJ1*, γ-synuclein (*SNCG*), and *ISLET1* are shown. Real-time PCR analysis was performed on total mRNA extracted from hESC-derived RGCs on D30. *BRN3B* is expressed at high levels (>1,000×) at D30, as compared with that analysed on D0. Each mRNA expression level was first normalised to HPRT1 expression and then compared to mRNA expression on D0. (**b**) Immunohistochemical detection of BRN3B, ATOH7, TUJ1, SNCG, and ISLET1 and double-staining of ATOH7 and TUJ1 on D30. Whole-mount staining was performed for TUJ1; serial section staining was performed for the other targets. BRN3B and SNCG staining is mostly visible at the peripheral margins of the attached OV, while ATOH7 and ISLET1 staining is also visible at the centre of the OV. ATOH7 stains the nucleus of the RGCs. Axons stained by TUJ1 emerges from ATOH7-positive cells. Scale bars, 100 μm. Scale bar for double-staining, 15 μm. Error bars, standard deviation.

To assess the presence of characteristic markers of RGCs (Fig. 1), the amount of mRNA, relative to D0, specific to each of the target genes, was calculated using the 2^−ΔΔCT^ method. The expression of mRNA was assessed by evaluating threshold cycle (CT) values. The CT values were firstly normalised to the expression level of hypoxanthine phosphoribosyl transferase 1 (HPRT1), and the relative amount of mRNA specific to each of the target genes was calculated using the 2^−ΔΔCT^ method. For evaluating the effects of chemical agents, the amount of mRNA relative to the amount of control mRNA was calculated. The sample size for all mRNA data was five (n = 5).

### Immunohistochemistry and histology

Immunostaining was performed as described previously^24^ using frozen sections or entire cells fixed in 24-well plates and 3.5-cm film-bottom dishes (Matsunami Glass). The stained cells were viewed with an IX71 inverted research microscope (Olympus) and a DeltaVision ELITE fluorescence microscope (CORNES Technologies). Frozen sections were prepared as previously described^24^. Hematoxylin and eosin staining of 3-μm-thick specimens was performed as previously described^24^. Growth cone immunostaining was performed by attaching OVs to 3.5-cm film-bottom dishes on D28, 1 day after administration of chemical agents, and conducting double-staining with phalloidin and GAP43 antibody. The primary antibodies and their respective dilutions used in this study are listed in Table 1, and negative controls for these antibodies are indicated in Fig. S1.

**Table 1:** Antibody list. The antibodies are used for immunohistochemistry.

	Gene Product	Supplier	Product Number	Dilution
1	BRN3B	Santa Cruz	sc-6026	1:50
2	ATOH7	Millipore	AB5694	1:50
3	ISLET1	Abcam	ab20670	1:50
4	SNCG	GeneTex	GTX110483	1:50
5	TUJ1	Sigma	T-5076	1:200
6	TAU	Cell Signaling Technology	#4019S	1:100
7	NFL	Cell Signaling Technology	#2837S	1:100
8	GAP43	Abcam	Ab75810	1:100

### Screening assays for neurotrophic and chemorepellent agents

Recombinant human NGF (R&D Systems), SEMA3A (R&D Systems), and human SLIT1 (R&D Systems), diluted in Dulbecco’s phosphate-buffered saline (DPBS, Life Technologies) were used as candidate agents with a potential to affect the axonal growth of hESC- and hiPSC-derived RGCs. DPBS was used as the control. These were added to RMM at final concentrations of 50, 100, or 200 ng/ml for NGF and SEMA3A, and 0.2, 1, 2, or 5 μg/ml for SLIT1. Further, SEMA3A and SLIT1 were supplemented on D27, at the time of attachment. For the supplementation assay of NGF, the induction protocol of RGCs was slightly modified, because the previously established protocol induced maximal growth of RGC axons. In particular, the supplementation of NGF was initiated on D24 without FBS or retinoic acid. On D27, attachment of floating EBs was achieved in the absence of FBS or BDNF^26^. Supplementation of these agents was continued until D30, 3 days after attachment, and the colonies were collected on D30.

### Local and sustained release of neurotrophic and chemorepellent agents from hydrogels

A quarter piece of hydrogel (Hydrogel #SP PI5, MedGel) was used as a carrier. Recombinant human NGF, diluted in reduced Matrigel (BD Bioscience), was used at 10 ng/ml, and SEMA3A, diluted in reduced Matrigel (BD Bioscience), was used at a concentration of 200 ng/ml, while recombinant human SLIT1 (R&D Systems), diluted in Matrigel, was used at concentrations of 5 µg/ml at a temperature of approximately 4 °C. Matrigel without the agents was used as the control. Each EB was adhered to the centre of a well in 24-well plates. Matrigel was absorbed by the piece of hydrogel, which was placed at the edge of each well of the 24-well plates, in front of the attached OVs on D29 (Fig. S2). On D31, 2 days after the administration of the respective agents, colonies were fixed and immunostained using neurofilament protein L (NFL) antibodies. Each experiment was repeated at least five times.

### Focal and sustained release of neurotrophic and chemorepellent agents from beads

Affi-gel Blue beads (Bio-Rad) were used as carriers for the sustained release of chemical agents. Beads were washed in DPBS and then incubated for 1 h at room temperature (20 °C) in solutions containing chemical agents at different concentrations. Recombinant human NGF (R&D Systems) and SEMA3A (R&D Systems) diluted in DPBS were used at a concentration of 200 ng/ml, and recombinant human SLIT1 (R&D Systems) diluted in DPBS was used at a concentration of 5 μg/ml. Beads without the agents were used as the controls. Beads were transplanted on D28, one by one, at the site near the base of attached OVs using forceps (Inami) and fine needles (TERUMO) under a microscope (Olympus). Because the filopodia of axons are away from the attached OV on D29, i.e., the day of starting administration of the agents by means of hydrogel, we transplanted the beads on D28 when the filopodia are located close to the OVs. Each experiment was repeated at least five times.

### Time-lapse observation of nerve pathfinding

A protocol similar to the one described for the local and sustained agent release assay was used to observe time-lapse analysis of axonal growth. A piece of hydrogel containing SLIT1 (5 μg/ml) diluted in Matrigel was placed in front of attached OVs on D29. Time-lapse observation was performed for 18 h in a STX Stage Top Incubator (TOKAI HIT) and imaged using an IX71 inverted research microscope (Olympus).

### Statistical Analysis

All statistical analyses were performed using PASW statistics 18 (IBM) software with values expressed as mean ± standard deviation (SD). Groups of measurements were compared using one-way analysis of variance (ANOVA) followed by Tukey’s honestly signify difference (Tukey-HSD) test for normally distributed variables, and Dunnett’s T3 test for variables of non-normal distribution. Probability values equal to or smaller than 5% were considered significant. In the current experiment, there was no non-parametric data.

## Results

### Generation of retinal ganglion cells from human embryonic stem cells

We attempted to generate RGCs from hESCs using the same protocol as used for the generation of RGCs from hiPSCs^24^. Real-time PCR revealed that characteristic RGC markers, such as *ATOH7* ^27^, *BRN3B* ^28^, *TUJ1*, γ-synuclein (*SNCG*)^29^, and *ISLET1* ^30^, were expressed more on D30 as compared with their expression on D0. In particular, the expression level of *BRN3B*—a highly specific marker for RGCs^28^—was increased more than 1000-fold (Fig. 1a). These results correspond to results reported for markers of hiPSC-derived RGCs^24^. Immunohistochemistry on D30 revealed that BRN3B-positive RGCs tended to be located on the peripheral margin of attached OVs. Similar findings were noted in SNCG, and ATOH7 and ISLET1-positive cells that were also detected at the margin and the centre of attached OVs. Staining of TUJ1 was evident at the radiating axons that developed from the attached OVs. Double-staining with ATOH7 and TUJ1 indicated that TUJ1-positive axons originated from ATOH7-positive RGCs. (Fig. 1b). These results demonstrated that RGCs can be generated from hESCs using the same protocol that is used to generate RGCs from hiPSCs. Furthermore, we confirmed that hESC-derived RGCs possess the same physiological functions, including axonal transport and action potentials, as those reported for hiPSC-derived RGCs^24^ (Fig. S3, and Supplementary Movie 1). Using puff application of 1 mM glutamate, we also observed an inward current and repetitive action potentials (Fig. S4).

Combining the results of our current and previous studies confirms that hESC- and hiPSC-derived RGCs expressed characteristic molecular and electrophysiological markers of RGCs. Therefore, hESC-derived RGCs were ready for use in the *in vitro* evaluation of the effects of neurotrophic and chemorepellent agents.

### Evaluation of the effects of nerve growth factor supplementation on retinal ganglion cells

**Figure 2:**
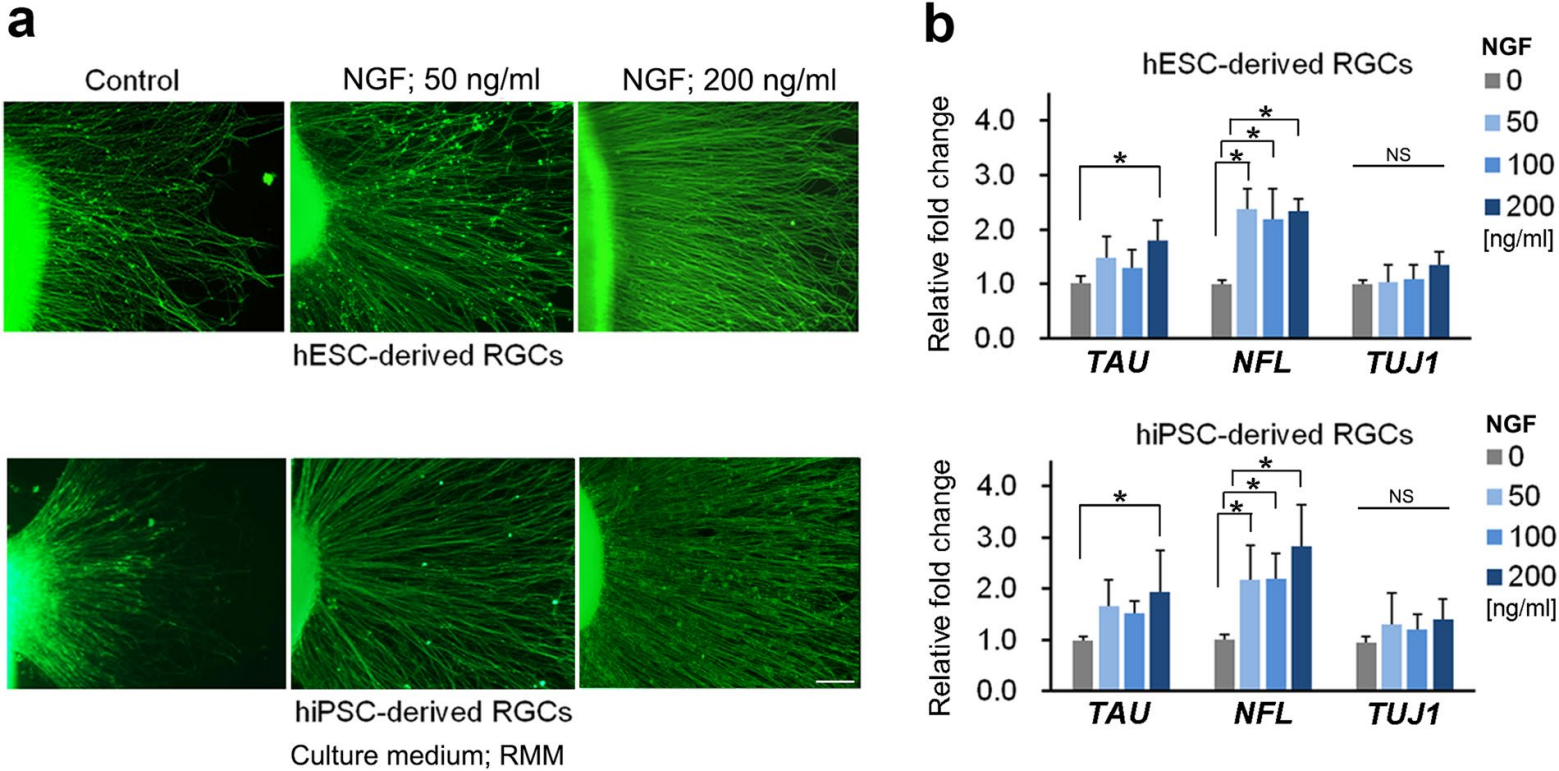
Effect of NGF supplementation on axonal growth of hESC- and hiPSC-derived RGCs. (**a**) Axonal growth of hESC- and hiPSC-derived RGCs was investigated by immunostaining of neurofilament protein L (NFL). Axonal growth of hESC- and hiPSC-derived RGCs is promoted by 50 and 200 ng/ml NGF supplementation from D24-30 (centre and right panels, respectively). The assessment from D24 is performed in retinal maturation medium (RMM). From D24 to D27, the embryoid bodies (EBs) are cultured in floating state and the EBs are attached to the dishes on D27, following 3 days’ adhesion culture. (**b**) Real-time PCR analysis of mRNA expression of axonal markers, *TAU*, *NFL*, and *TUJ1* (n = 5). Expression of *NFL* is significantly upregulated at all concentrations of NGF supplementation (ANOVA, Dunnett’s T3 test, p ≤ 0.05). *TAU* expression is significantly increased only for 200 ng/ml NGF supplementation (ANOVA, Dunnett’s T3 test, p ≤ 0.05). No significant increase of *TUJ1* expression is detected for all concentrations of NGF supplementation. There is no different reaction between hESC- and hiPSC-derived RGCs. Scale bar, 100 μm. Error bars indicate ± standard deviation (SD). Each column shows an average value for the studied samples. The sample size for all mRNA data is five (n = 5). NS, not significant.

We first investigated the availability of hESC- and hiPSC-derived RGCs in order to evaluate the effects of totally and locally administrated growth factor. NGF (a growth factor regulating the growth and survival of nerve cells^31^) was added to floating aggregates in the culture media on D24, at concentrations of 0, 50, 100, and 200 ng/ml. After attachment to the well on D27, supplementation was continued for another 3 days. Immunohistochemistry by NFL-staining revealed a starkly increased number and length of axons following the supplementation of NGF to each well (Fig. 2a). Growth of axons of hESC- and hiPSC-derived RGCs was significantly promoted by 200 ng/ml NGF supplementation, and there was no difference between hESC- and hiPSC-derived RGCs (Fig. S5f).

These results were supported by real-time PCR data of the axonal markers *NFL* ^32^ and *TAU* ^33^. In RGCs generated from hESCs and from hiPSCs, the *NFL* expression levels were upregulated with NGF supplementation at concentrations of 50, 100, and 200 ng/ml, as compared with samples from control conditions (ANOVA, Dunnett’s T3 test, P ≤ 0.05). The expression pattern did not change in a dose-dependent manner. The expression of *TUJ1* tended to be upregulated by NGF supplementation; however, the increase was not statistically significant (Fig. 2b). The upregulation of NFL by NGF supplementation was clearly inhibited by K252, which is an established NGF receptor antagonist (Fig. S6).

### Evaluation of the effects of SEMA3A supplementation on retinal ganglion cells

**Figure 3:**
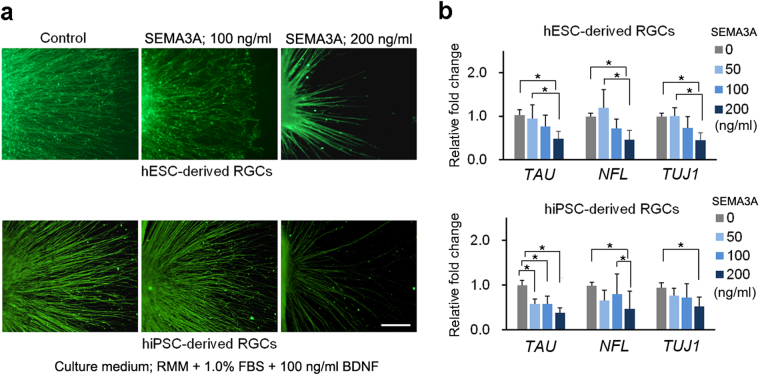
Effect of SEMA3A supplementation on axonal growth of hESC- and hiPSC-derived RGCs. (**a**) Axonal growth of hESC- and hiPSC-derived RGCs is observed by immunostaining of NFL. Axonal growth of hESC- and hiPSC-derived RGCs is starkly inhibited by 100 and 200 ng/ml SEMA3A supplementation from D27−30 (centre and right panels, respectively). The assessment from D27 is performed under RMM supplemented with 1.0% FBS and 100 ng/ml BDNF. (**b**) Real-time PCR analysis of mRNA expression of axonal markers, *TAU*, *NFL*, and *TUJ1*. In both hESC- and hiPSC-derived RGCs, the expression of, *TAU*, *NFL*, and *TUJ1* appears to decrease in a dose-dependent manner. Expression levels of all markers are significantly decreased at 200 ng/ml SEMA3A supplementation (ANOVA, Tukey-HSD test, p ≤ 0.05). Scale bar, 100 μm. Error bars indicate±SD. Each column shows an average value for the studied samples. The sample size for all mRNA data is five (n = 5). NS, not significant.

We investigated the usefulness of our hESC- and hiPSC-derived RGCs for evaluation of the effects of totally and locally administrated suppression factors. SEMA3A, which guides axonal growth by chemorepulsion via growth cone collapse^34^, was added to each well of hESC- and hiPSC-derived RGCs on D27, and supplementation was continued for 3 days at concentrations of 0, 50, 100, or 200 ng/ml. Immunohistochemistry using NFL staining revealed that the number and length of axons markedly decreased after SEMA3A supplementation (Fig. 3a). Growth of axons of hESC- and hiPSC-derived RGCs are significantly decreased by 200 ng/ml SEMA3A supplementation, and there was no difference between hESC- and hiPSC-derived RGCs (Fig. S5d).

These results were supported by RT-RCR data of the axonal markers *NFL, TAU*, and *TUJ1*. In RGCs generated from hESCs and from hiPSCs, the expression level of *NFL* was significantly downregulated at a concentration of 200 ng/ml SEMA3A, compared with the control (ANOVA, Tukey-HSD test, P ≤ 0.05). The downregulation tended to be dose-dependent. Expression of *TAU* and *TUJ1* was significantly downregulated at a concentration of 200 ng/ml SEMA3A (ANOVA, Tukey-HSD test, P ≤ 0.05). Expression levels tended to be lower relative to control levels for all three concentrations tested; however, this trend was not statistically significant for concentrations of 50 and 100 ng/ml (Fig. 3b).

### Evaluation of the effects of SLIT1 supplementation on retinal ganglion cells

**Figure 4:**
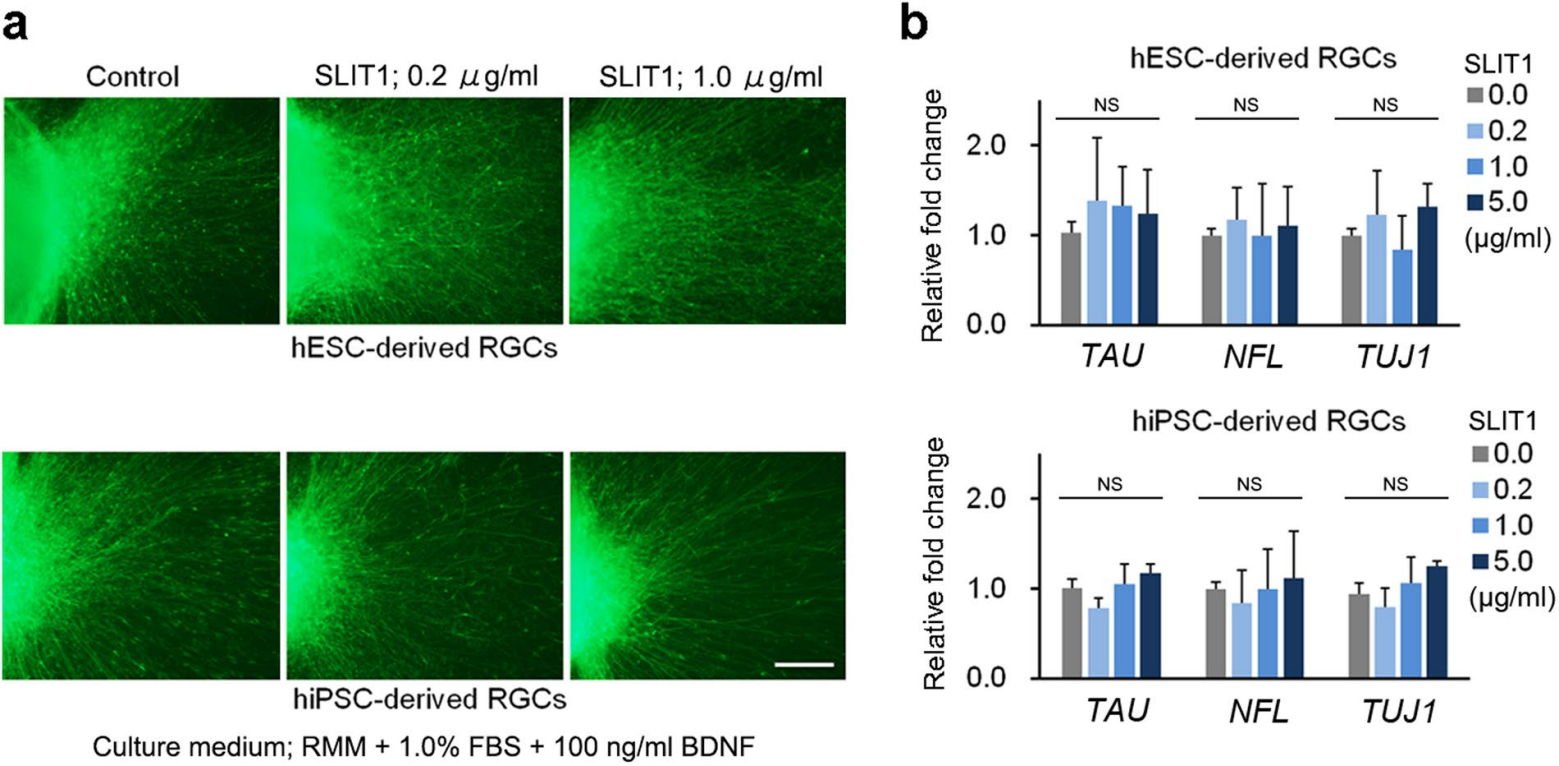
Effect of SLIT1 supplementation on axonal growth of hESC- and hiPSC-derived RGCs. (**a**) Axonal growth of hESC- and hiPSC-derived RGCs is observed by immunostaining of NFL. In the control, hESC- and hiPSC-derived RGCs axons grow radially and straight (left panels). In contrast, the growth pattern of axons after SLIT1 supplementation is complex. Axonal growth of hESC- and hiPSC-derived RGCs is not inhibited by addition of 0.2 and 1.0 µg of SLIT1 supplementation from D27−30 (centre and right panels, respectively). However, axonal paths are disturbed (centre and right panels). The assessment at D27 is performed in RMM supplemented with 1.0% FBS and 100 ng/ml BDNF. (**b**) Real-time PCR analysis of mRNA expression of axonal markers, *TAU*, *NFL*, and *TUJ1*. Expression levels of axonal markers are not influenced by SLIT1 supplementation at any of the concentrations tested. Scale bar, 100 μm. Error bars indicate ± SD. Each column shows an average value for the studied samples. The sample size for all mRNA data is five (n = 5). NS, not significant.

We investigated the usefulness of our hESC- and hiPSC-derived RGCs for evaluation of the effects of a totally and locally administered nerve bending factor during pathfinding. SLIT1, which plays an important role in axonal guidance by sending repulsive signals to the axon^35^, was added to each well of hESC- and hiPSC-derived RGCs on D27 at concentrations of 0.0, 0.2, 1.0, and 5.0 μg/ml. Immunohistochemistry using NFL staining revealed no inhibition of axonal growth, as compared with the control. However, we observed a change in axon morphology; rather than growing straight, several axons grew windingly, indicating axon misguidance (Fig. 4a). The grown axonal length of hESC- and hiPSC-derived RGCs was not decreased by supplementation with 5 μg/ml SLIT1 (Fig. S5e). These results were supported by RT-RCR data of the axonal markers *NFL*, *TAU* and *TUJ1*; expression levels tended to be similar to those of the control at all concentrations (Fig. 4b).

### Evaluation of the effect of local and focal sustained release of nerve growth factor to retinal ganglion cells

**Figure 5:**
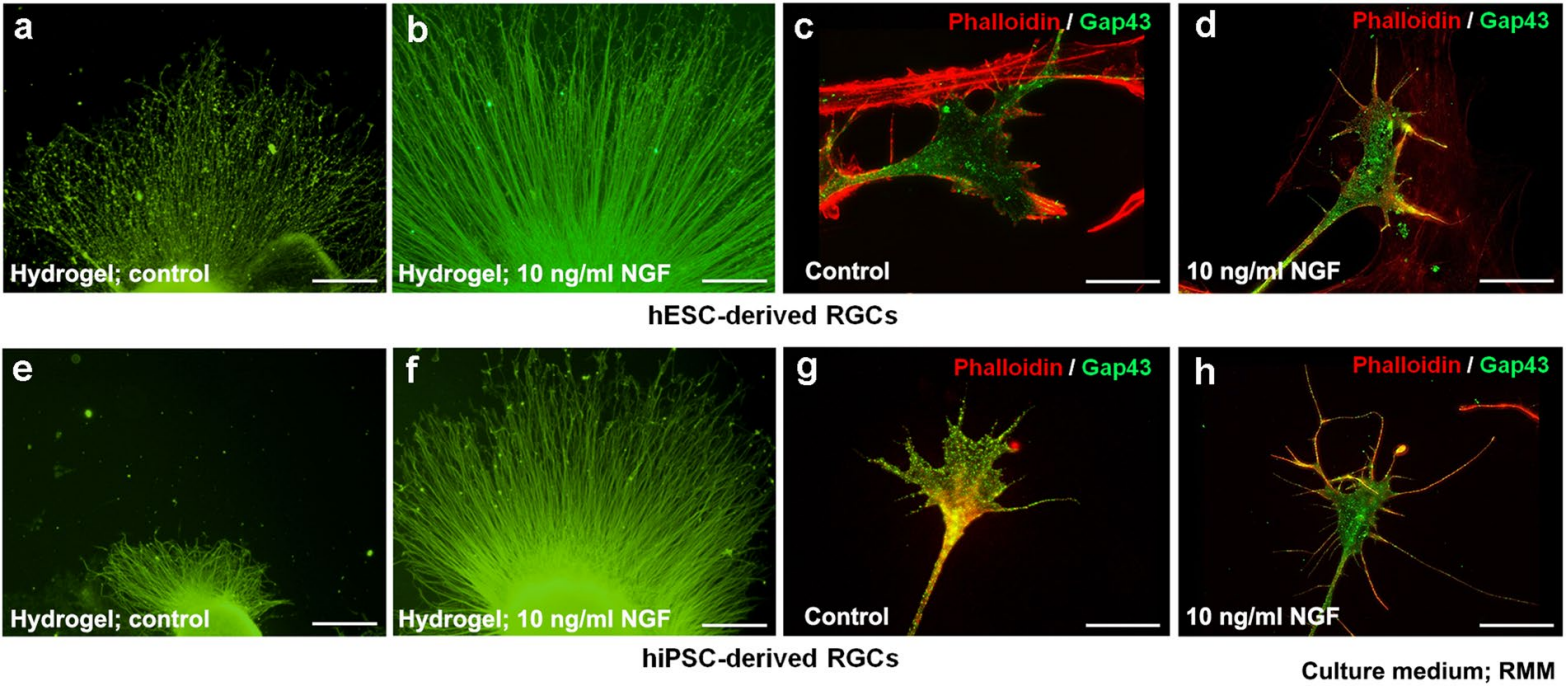
Effect of local sustained release of NGF (10 ng/ml) from hydrogels placed in front of the attached OVs. Local sustained release of NGF using hydrogel promoted axonal growth, stained by NFL in hESC- and hiPSC-derived RGCs ((**b**) and (**f**), respectively, magnification 40x), as compared with controls ((**a**) and (**e**), respectively). The shape of the filopodia of hESC- and hiPSC-derived RGCs ((**d**) and (**h**), respectively) was slightly promoted by NGF release, as compared with controls ((**c**) and (**g**), respectively), as evident from the staining of phalloidin and GAP43. The assessment from D24-D31 is performed in RMM. Each experiment was repeated at least five times. Scale bars in (**a**), (**b**), (**e**) and (**f**), 100 μm. Scale bars (**c**), (**d**), (**g**) and (**h**), 10 μm.

We next investigated the effects of local and sustained delivery of NGF from hydrogel or focally transplanted beads. A piece of hydrogel containing NGF (10 ng/ml) was placed inside the well in front of the OV 2 days after attachment, on D29, and culture was continued for 2 more days. NGF hydrogel promoted axonal growth of hESC- and hiPSC-derived RGCs (Fig. 5b,f, respectively), as compared with controls (Fig. 5a,e, respectively). Phalloidin and GAP43 staining confirmed that filopodia of hESC- and hiPSC-derived RGCs (Fig. 5c,g, respectively) were normally organised and their growth was slightly promoted after NGF treatment (Fig. 5d,h, respectively). Beads containing NGF (200 ng/ml) were transplanted beside the OV 1 day after attachment, but no obvious effect was observed (Fig. S7).

### Evaluation of the effect of local and focal sustained release of SEMA3A to retinal ganglion cells

**Figure 6:**
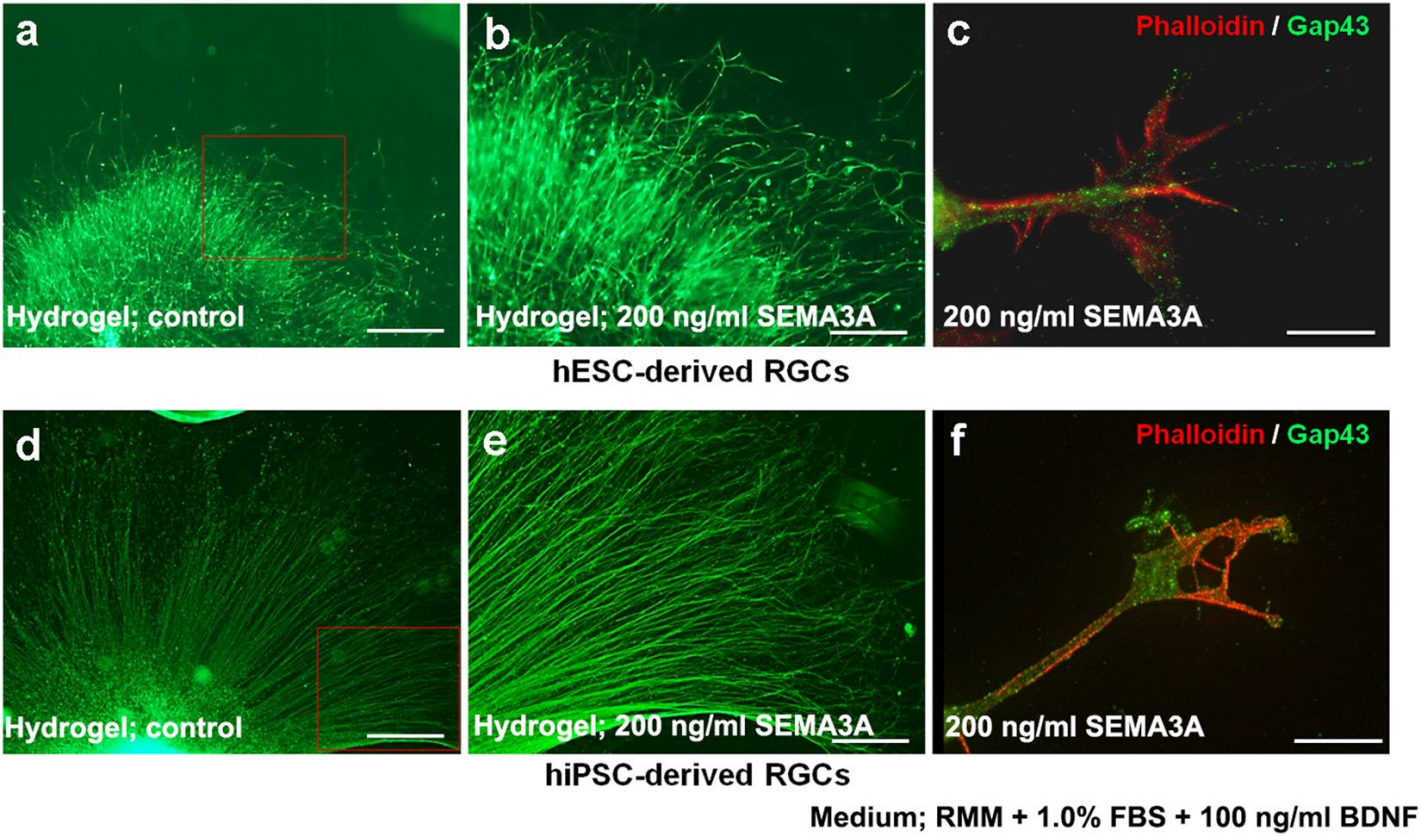
Effect of locally sustained release of SEMA3A (200 ng/ml) from hydrogels placed in front of attached OVs. Locally sustained release of SEMA3A using hydrogel inhibited anterior axonal growth of hESC- and hiPSC-derived RGCs ((**a**) and (**d**), respectively, magnification 40x). In magnified image, most axons are clearly repelled by SEMA3A release ((**b**) and (**e**), magnification 100x: squares in (**a**) and (**d**), respectively). The filopodia of hESC- and hiPSC-derived RGCs ((**c**) and (**f**), respectively) have collapsed owing to SEMA3A release, as evident from phalloidin and GAP43 staining. The assessment from D27 is performed in RMM supplemented with 1.0% FBS and 100 ng/ml BDNF. Each experiment was repeated at least five times. Squares in (**a**) and (**d**) correspond to (**b**) and (**c**), respectively. Scale bars in (**a**) and (**d**), 100 μm. Scale bars in (**b**) and (**e**), 40 μm. Scale bars (**c**) and (**f**), 10 μm.

We next investigated the effects of local and sustained delivery of SEMA3A from hydrogel or focally transplanted beads. A piece of hydrogel containing SEMA3A (200 ng/ml) was placed inside the well in front of the OV 2 days after attachment on D27, and culture was continued for 2 more days. SEMA3A-hydrogel locally inhibited elongation of axons, as revealed by immunohistochemistry, indicating the chemorepellent effects of SEMA3A on axonal growth (Fig. 6a,b,d,e). The morphology of the filopodia was not maintained and collapsed after SEMA3A supplementation (Fig. 6c,f). Effects were similar for hESC- and hiPSC-derived RGCs. Beads containing SEMA3A (200 ng/ml) were transplanted beside the OV 1 day after attachment, but no obvious effect was observed (Fig. S8).

### Evaluation of the effect of local and focal sustained release of SLIT1 to retinal ganglion cells

**Figure 7:**
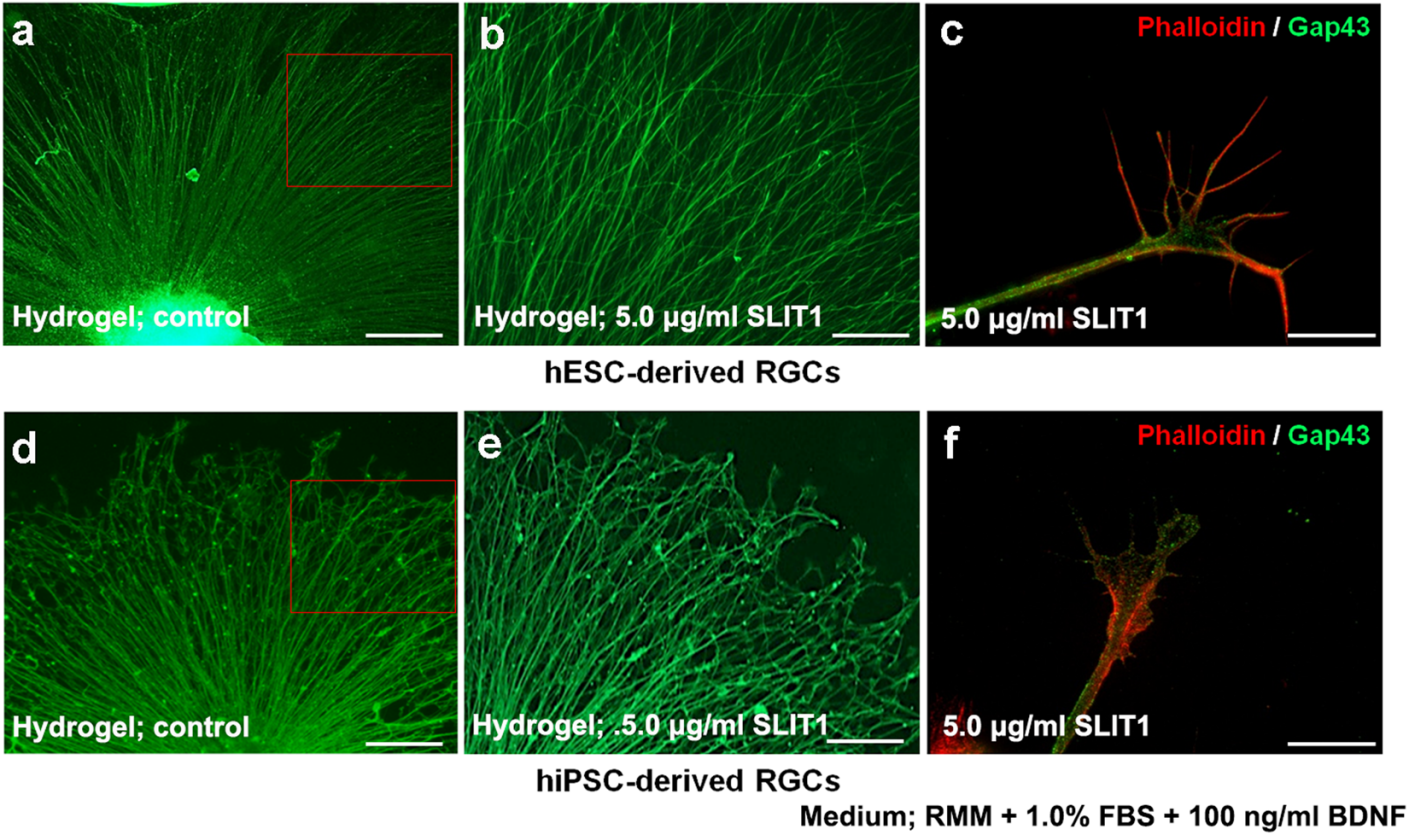
Effect of locally sustained release of SLIT1 (5μg/ml) from hydrogels placed in front of the attached OVs. Locally sustained release of SLIT1 using hydrogel does not affect axonal growth of hESC- and hiPSC-derived RGCs ((**a**) and (**d**), respectively, magnification 40x). Only a subset of axons is repelled by SLIT1 release ((**b**) and (**e**), magnification 100x: squares in (**a**) and (**d**), respectively), while other axons remain unaffected. Filopodia of hESC- and hiPSC-derived RGCs ((**c**) and (**f**), respectively) have collapsed in response to SLIT1 release, as evident from phalloidin and GAP43 staining. The assessment from D27 is performed under RMM supplemented with 1.0% FBS and 100 ng/ml BDNF. Each experiment was repeated at least five times. Squares in (**a**) and (**d**) correspond to (**b**) and (**e**), respectively. Scale bars in (**a**) and (**d**), 100 μm. Scale bars in (**b**) and (**e**), 40 μm. Scale bars in (**c**) and (**f**), 10 μm.

**Figure 8:**
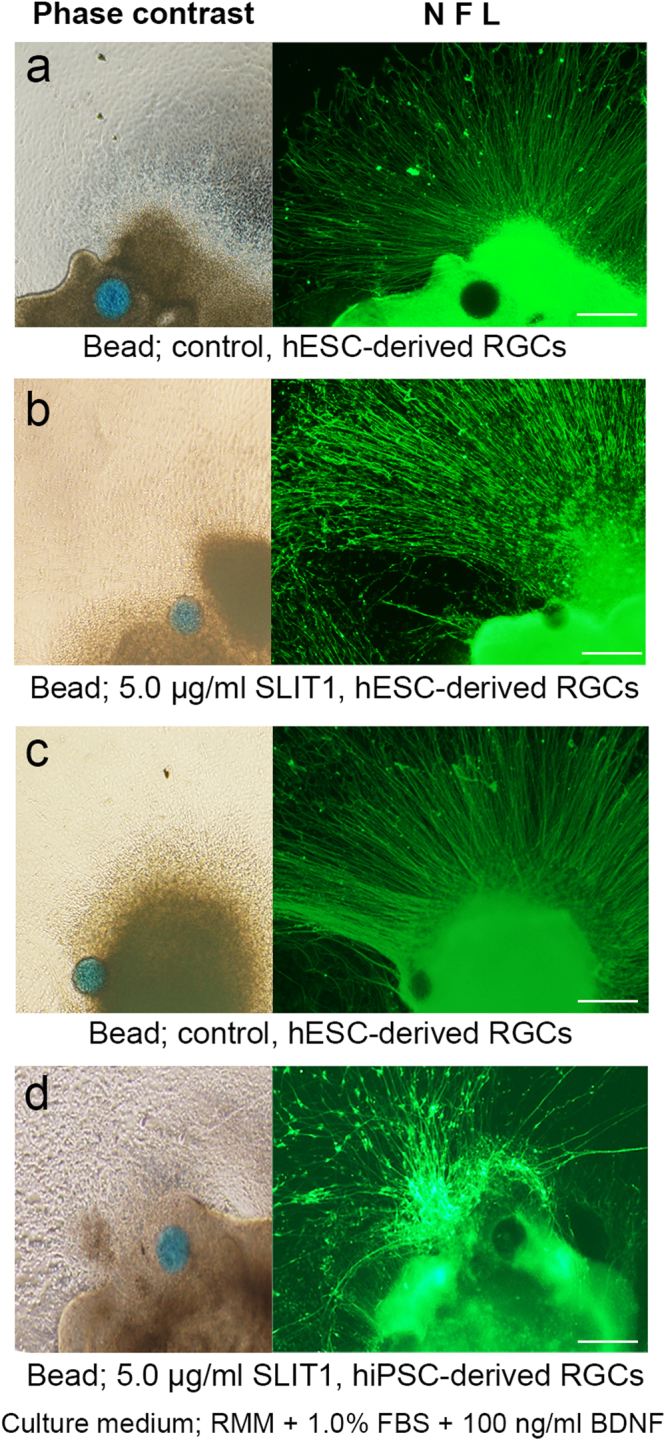
The effect of focally sustained release of SLIT1 on pathfinding of axons derived from hESC- and hiPSC-derived RGCs. Phase-contrast images showing SLIT1-releasing beads (blue-coloured) placed next to the bottom of attached OV, and corresponding immunohistochemistry for the axons of the RGCs by NFL staining. (**a,c**) The paths of the axons of hESC- and hiPSCs- derived RGCs ((**a**) and (**c**), respectively) stained by NFL radiate straight from the attached OV, even though the bead is located close by. (**b**,**d**) Compared with the control, the axons of hESC- and hiPSC-derived RGCs ((**b**) and (**d**), respectively) are repelled by SLIT1 release from the bead, and axonal paths are not straight, but winding, and avoid the beads. The assessment from D27 is performed in RMM supplemented with 1.0% FBS and 100 ng/ml BDNF. Each experiment was repeated at least five times. Scale bars, 100 μm.

We next investigated the effects of local and sustained delivery of SLIT1 from hydrogel or focally transplanted beads. A piece of hydrogel containing SLIT1 (5 μg/ml) was placed inside the well in front of the OV 2 days after attachment on D27; the culture was then continued for 2 more days. SLIT1-hydrogel did not inhibit axonal growth. However, a subset of axons progressed as if avoiding the hydrogel (Fig. 7a,b,d,e), in both hESC- and hiPSC-derived RGCs, which was calculated using hESC-derived RGSs (Fig. S9). The morphology of the lamellipodia, rather than filopodia, was not maintained, and collapsed following SLIT1 supplementation (Fig. 7c,f). Similar effects were noted for hESC- and hiPSC-derived RGCs. Beads containing SLIT1 (5 μg/ml) were transplanted beside the OVs (Fig. 8) 1 day after attachment. In RGCs derived from both hESCs and hiPSCs, axons located close to the beads formed a bend, avoiding the area close to SLIT1-beads (Fig. 8b,d, respectively), compared with the control (Fig. 8a,c, respectively).

### *In vitro* time-lapse observation of the effects of SLIT1 on nerve pathfinding

In the above experiments, SLIT1 acted as a chemorepellent for axonal growth in hESC- and hiPSC-derived RGCs. Therefore, real-time axonal pathfinding was imaged *in vitro* during local and sustained release of SLIT1 from hydrogels placed in front of attached OV for 18 h. We found that a subset of axons actively regressed while other axons actively changed their path to avoid the SLIT1-hydrogel both in hESC- and hiPSC-derived RGC cultures (Supplementary Movie 2).

## Discussion

In the current study, we first demonstrated that RGCs could also be generated from hESCs using the same protocol as for the generation of RGCs from hiPSCs. Minute differences between these two types of cells have been reported previously, such as variations in the expression patterns of non-coding RNAs^36,37^. However, organogenesis of the islets of the pancreas, nephrons, and, the pituitary gland has been reported from both hiPSCs, and hESCs. In accordance with these reports, most of the RGC markers tested, including by means of assays of genes expression, protein expression, axonal transport, and action potential firing, were similar for RGCs derived from hESCs and from hiPSCs^24^. Therefore, RGCs can be generated from both stem cell types, and the RGCs should be equally useful as a model of human RGCs.

The technique for the *in vitro* generation of RGCs from hiPSCs produced these cells with 90% efficacy and cells remained stable for around 50 days^24^; RGCs were similarly generated from hESCs (data not shown). We then evaluated the effects of neurotrophic and chemorepellent agents on the RGCs derived from hESCs and hiPSCs, generated in this study. To assess the general effect of each of these agents, we added them to each culture well, and to observe the local effects of the agents, we used hydrogel and focally transplanted beads as carriers for these agents. The hydrogel was placed in front of the attached OV, which enabled us to observe the nerve pathfinding in the context of the sustained release agents from the gel. On the other hand, focally transplanted beads enabled us to observe the effect of the agents on axonal pathfinding of the RGCs released from the inner portion of primordium of the retina, i.e., the attached OV.

We initially examined the possible application of the RGCs from hESCs and hiPSCs for the assessment of neurotrophic agents *in vitro* using NGF as a neurotrophic factor. NGF supplementation to the entire dish promoted axonal growth in RGCs, even at the lowest concentration tested (50 ng/ml). The mRNA expression levels of axonal structure-related genes supported this result. When release of 10 ng/ml NGF was locally sustained using hydrogel, RGC axon growth toward the hydrogel was also promoted. NGF is an important growth factor regulating growth and survival of nerve cells through tropomyosin receptor kinase A (TrkA)^31^ and p75NTR receptor binding. TrkA positively regulates cell survival^38,39^; in contrast, p75NTR induces nerve cell death by apoptosis^40,41^. Some researchers have demonstrated that NGF treats glaucoma effectively^42,43^, while others have published negative results^44^. This ambiguity can probably be explained by the bilateral effect of NGF^45^. Axons were successfully promoted by 200 ng/ml NGF supplementation to the entire dish, but the promotion was observed at a lower dose (10 ng/ml NGF) in locally placed hydrogel assay, which may be because of the positive and negative effects of NGF on RGC survival.

For the NGF assay, we first applied NGF to the dish from D27, the day of attachment, in line with our original induction method, but no significant axonal promotion was observed (data not shown). We considered that the effects of NGF may have been masked by the BDNF contained in the culture medium^26,46^ and by other neurotrophic factors that may have been present in FBS. We therefore excluded BDNF and FBS from the cultures from D24, and added NGF. This *in vitro* assessment system enabled us to analyse the effects of NGF on the promotion of axons of RGCs precisely *in vitro*.

Secondly, we examined the effects of the chemorepellent agents SEMA3A and SLIT1 on RGCs. Supplementation of SEMA3A to the whole dish inhibited axonal growth of RGCs in a dose-dependent manner. Inhibition of axonal growth was confirmed both by immunohistochemical analysis and by analysis of the mRNA expression levels of axonal makers *NFL*, *THJ1*, and *TAU*. In contrast, SLIT1 affected neither axonal elongation nor expression levels of axonal markers. Judging from immunohistochemical analysis, SLIT1 only seemed to affect pathfinding of some axons, resulting in complex axonal elongation patterns. In further experiments on local sustained release of SEMA3A and SLIT1, most axons seemed to be repelled by SEMA3A release, in contrast to a subset of axons affected by SLIT1 release. Although growth cone collapse was directly observed by double-staining of phalloidin and GAP43, both under SEMA3A and SLIT1 supplementation, these results may indicate that SEMA3A indiscriminately induces collapse of axonal cones, while SLIT1 only induces the collapse of specific cones expressing the roundabout (Robo) receptor.

Semaphorins and SLITs are highly conserved proteins in both vertebrates and invertebrates^47,48^; members of both of these families achieved chemorepulsion via growth cone collapse, in concert with neuropilins 1/2 and A-plexins^49^ and Robo receptor^35,47^, respectively. In comparison with the semaphorins, which also play a crucial role in the development and morphogenesis of various organs^34,50–52^, SLITs are more locally expressed in the central nervous system, including in the eye^53,54^. Expression of SEMA3A has been detected in the RGCs of rat and zebra fish retina^55,56^, and RGC growth was repelled by SEMA3A in Xenopus RGCs^57^. Expression of SEMA3A has also been detected near the optic chiasm and in the optic tectum of zebra fish^56^. SLIT/Robo signalling also plays a crucial role in navigating RGC axons in the developing retina. SLIT1 and −2 are expressed in the RGC itself and in the inner nuclear layer, and they determine the polarity of axons in the nerve fibre layer^58^. SLITs are also expressed at the optic chiasm and the optic tract and act as chemorepellent factors. Although both SAMA3A and SLIT1 appear to influence RGC development in a similar fashion, SEMA3A seems to have more impact on RGC development than SLIT1, according to the results of the current *in vitro* experiment. Further experiments are needed to determine why different results were obtained for the two molecules. Nonetheless, this study demonstrates that the *in vitro* assessment system developed here significantly facilitates evaluation of the effects of these molecules on human RGCs.

In the assay for screening of chemical agents, the effect of each axon guidance factor on RGCs was clearly assessed, in which the length of axons and its molecular markers changed, depending on the respective dosages. Local administration of those axon guidance factors further confirmed the effect of each axon guidance facto on the axons; NGF promoted axonal elongation in the direction of the agent and SEMA3A and SLIT1 had a chemorepellent effect on axons. Direct observation of growth cones revealed that these phenomena were achieved through changes in the shape of the filopodia. These novel *in vitro* observations were possible only because the RGCs established in this study developed elongated axons with numerous growth cones; similar observations would have been impossible using RGCs isolated from animal and human retinas. It was possible to culture a large number of RGCs using our protocol, and it may be possible to enhance the method in future, for instance, through pharmaceutical analysis of RGCs supplemented with various candidate drugs, using molecular experiments, including microarrays.

In contrast to total and local axon guidance factor administration, focal transplantation of beads containing each axon guidance factor allows more precise observation of the effects on nerve pathfinding. To investigate focal effects of NGF, SEMA3A, and SLIT1 on axonal pathfinding of RGCs, we transplanted beads containing the respective molecules close to the attached OVs from which the RGCs developed. In our experiments, focal sustained release of NGF and SEMA3A had little impact on axonal pathfinding, while focal sustained release of SLIT1 resulted in the rerouting of RGC axons. As discussed above, SLIT1 is expressed at the inner nuclear layer of the developing retina, and plays a crucial role in determining the polarity of axons, by restricting axon progression into the nerve fibre layer^59^. In addition, SLITs are expressed in the optic chiasm, causing Robo-expressing growth cone collapse and resulting in commissural axons^60^. In the current study, SLIT1-containing beads were transplanted at the time of initial axonal development. Therefore, focal sustained SLIT1 possibly mimics the phenomenon *in vitro*.

Time-lapse observation for monitoring SLIT1-induced axon repulsion was performed with the use of a locally sustained axon guidance factor system. Our analysis revealed that different axons reacted differently to SLIT1-containing hydrogel, in which some growing axons changed path and avoided the SLIT1-containing hydrogel, while others regressed. In the developing retina, primitive RGCs develop into several types of RGCs and 90% of RGCs underwent apoptosis^61^. RGCs generated *in vitro* by our system could mimic the *in vivo* developing RGCs. Thus, real-time observation of such cultures might aid our understanding of unknown developmental events of RGCs *in vitro*.

In the current and in a previous study, we established a method for generating human RGCs derived from hESCs and hiPSCs. In this study, we have developed an *in vitro* evaluation system for human RGCs that allows analysis of the effects of biomolecules *in vitro* and that may be useful for potential drug discoveries. We confirmed that the general effects of the agents were analysable through supplementation of each well. The nerve pathfinding and the growth cone were observed by locally used hydrogel and Affi-Gel blue. Chemorepellent factors were easier to analyse than neurotrophic factors. In addition, we established a real-time observation system for the human RGCs *in vitro*, in which the physiological selection process of RGCs was observed.

It has been difficult to obtain live, human neuronal cells, particularly RGCs, to date. However, due to the differentiation method we established in this study and to the methods other groups have developed^62–64^, the availability of human RGCs may now be significantly improved. Gill *et al*. developed stepwise retinal differentiation of hESCs, from which RGCs are selected by the MACS isolation method, by labelling THY1.1. Although these RGCs have long axons and physiological function, as do ours, the axons seems to be heterogeneous^64^. Sluch *et al*. also attempted to obtain purified RGCs derived from CRISPR-engineered hESCs, in which axons of the RGCs elongated through nanofibre matrices, and concluded that forskolin may be a potent promoter of RGC differentiation^63^. Compared with the previous reports, our system has a limitation regarding purification or isolation of RGCs, because they are accompanied by several cells inside the attached OVs and EBs. On the other hand, a remarkable advantage of our method for generating RGCs, which is achieved only by co-culture of EBs and OVs, is that it obtains uniform, straight-radiating, abundant, and long axons in RGCs from the attached OVs. These enriched axons allowed us to observe the physiology of the axons of human RGCs in real-time and to analyse the effects of neuroactive agents on RGCs, including the promotion and inhibition of axons and their pathfinding, and the effects of neuroactive agents applied at different times, concentrations, and combinations. Until fairly recently, animal experiments and clinical trials have been necessary for the exploration of new drugs, including neuroprotective agents, because human cells were difficult to obtain, and thus the effect of the drugs on human cells could not be determined. The current approach for using RGCs derived from hESCs and hiPSCs for developing new drugs is costly and time-consuming, and has prevented the speedy exploration of new drugs. The potential drug screening system that we propose may shortcut the process.

The generation of iPSCs by reprogramming patient tissue is now a routine procedure^65^ and new drugs have been explored *in vitro* for the treatment of diseases, such as Parkinson’s and Alzheimer’s disease, which are resistant to medication; this is an obvious advantage of using iPSCs. For example, using cortical neurons derived from fibroblasts of patients with autosomal dominant or sporadic Alzheimer’s disease, docosahexaenoic acid has been demonstrated to be effective in a subset of patients^66^. Forebrain neurons derived from patients with Parkinson’s disease have been used to screen potential new drugs. In these screenings, Nedd4, and NAB2 were identified as candidate drug molecules^67^. Our method of generating human RGCs, with their characteristic axons, provides a potential system for the *in vitro* screening of drugs that may have neuroprotective and neuro-regenerative functions in the human RGC; such screening is already underway in our laboratory. The system presented in the current study may aid our understanding of the physiological development of RGC axons and enable us to develop new drugs that would significantly improve the quality of life of patients with treatment-resistant optic neuropathies.

## Electronic supplementary material

Supplementary information

Supplementary Video1

Supplementary Video2
