# Supplementary material for: Effects of neuroactive agents on axonal growth and pathfinding of retinal ganglion cells generated from human stem cells
Source: Sci Rep. 2017 Dec 1;7:16757. doi: 10.1038/s41598-017-16727-1 (PMC5711798; doi:10.1038/s41598-017-16727-1)
Supplement: Supplementary file 1 — Supplementary information [file 41598_2017_16727_MOESM1_ESM.pdf]

Effects of neuroactive agents on axonal growth and pathfinding of retinal ganglion cells generated from human stem cells

Tadashi Yokoi[1], M.D., Ph.D.; Taku Tanaka[1], Ph.D.; Emiko Matsuzaka[1], Ph.D.; Fuminobu Tamalu[2], Ph.D.; Shu-Ichi Watanabe[2], M.D., Ph.D.; Sachiko Nishina[1], M.D., Ph.D.; and Noriyuki Azuma[1], M.D., Ph.D.

[1] Department of Ophthalmology and Laboratory for Visual Science, National Centre for Child Health and Development, Tokyo, Japan

[2] Department of Physiology, Faculty of Medicine, Saitama Medical University, Saitama, Japan

Supplementary Methods

Axonal transport observation

The time series images of anterograde axonal transport was generated as described previously[1] by the injection of Alexa Fluo-555-conjugated cholera toxin subunit B (Life Technologies) into the centre of attached OVs, followed by imaging the Alexa Fluo-555-conjugated cholera toxin subunit B using an IX71 inverted research microscope (Olympus).

Electrophysiological recording

Whole-cell patch-clamp recordings were performed as previously described[1]. Briefly, EBs were attached and cultured on mixed cellulose ester filter paper (0.2-µm pore size; ADVANTEC, Houston, TX) from D27. Whole-cell patch-clamp recordings were performed on RGCs located at the peripheral portion of attached OVs. RGCs exhibited repetitive action potentials. Recording pipettes (6–8 MW Mrding pipettes (6e action plar solution, as described previously[1]. The morphology of the recorded cells was visualised using Lucifer Yellow (LY). Liquid junction potentials (-11 mV) were corrected and R_s was compensated at 40%. Cells with R_s > 50 MW0 M Ronot included in the analysis. Average membrane capacitance was 8.3 ± 3.8 pF in hESC-derived

RGCs (n = 6). Current and voltage data were acquired using pCLAMP 9.2 software and saved on a custom-built personal computer (Physio-Tech, Tokyo, Japan). Analyses were performed with Clampfit 9.2 (Molecular Devices, Sunnyvale, CA) and OriginPro 2015 (OriginLab, Northampton, UK). Images of LY-filled cells were captured using a high-gain colour camera (HCC-600; Flovel, Tokyo, Japan) and saved using INFO.TV Plus software (Infocity, Gandhinagar, India). All data are presented as mean ± SD.

Local application of glutamate

We used a puffer pipette whose diameter was the same as the recording pipette (» 1 μm) for local application of glutamate to the recorded RGCs. The puffer pipettes were filled with an external solution containing (in mM): 135 NaCl, 3 KCl, 2.5 CaCl2, 1 MgCl2, 10 glucose, 10 HEPES, and 1 glutamate (pH 7.4) and positioned near the soma of recorded RGCs. We applied a pressure pulses continuously (10 kPa) via an electromagnetic valve (M5136-NB-M5; CKD, Japan) which was controlled using pCLAMP 9.2 software.

Supplementary Results

Physiological function of retinal ganglion cells generated from human embryonic stem cells

Furthermore, we confirmed that hESC-derived RGCs possess the same physiological functions as those reported for hiPSC-derived RGCs[1]. Firstly, we tested axonal transport in the axons apparent on D30. Anterograde axonal transport was assayed by injecting Alexa Fluor-conjugated cholera toxin B into the central portion of the attached OVs. Within 60 min, cholera toxin B had been transported through the axon by anterograde transport (Fig 2a and Supplementary Movie 1). Secondly, we evaluated whether hESC-derived RGCs were able to fire action potentials. We selected RGCs on the peripheral margin of attached OVs for whole-cell patch-clamp recordings (Fig. 2b). A representative RGC showing a long axon process is shown in Figure 2c. The sample of cells we recorded from the generated repetitive action potentials in response to current injection in current-clamp mode is shown in Figure 2d. Resting membrane potential was determined to be −67 ± 15 mV and the amplitude of the first action potential was recorded as 58 ± 14 mV (n = 6). Cells that generated action potentials exhibited tetrodotoxin-sensitive (TTX-sensitive) Na currents with maximum amplitudes of 958 ± 355 pA (n = 6), followed by outward currents (Fig. 2e). Using puff application of 1 mM

glutamate, we also observed an inward current of 28 ± 29 pA (n = 5) and repetitive action potentials with an amplitude of 69 ± 9.9 mV (n = 5) (Fig. S3). Combining the results of our current and previous studies confirms that hESC- and hiPSC-derived RGCs expressed characteristic molecular and electrophysiological markers of RGCs. Therefore, hESC-derived RGCs were ready for use in the *in vitro* evaluation of the effects of neurotrophic and chemorepellent agents.

Supplementary Figures

Figure S1

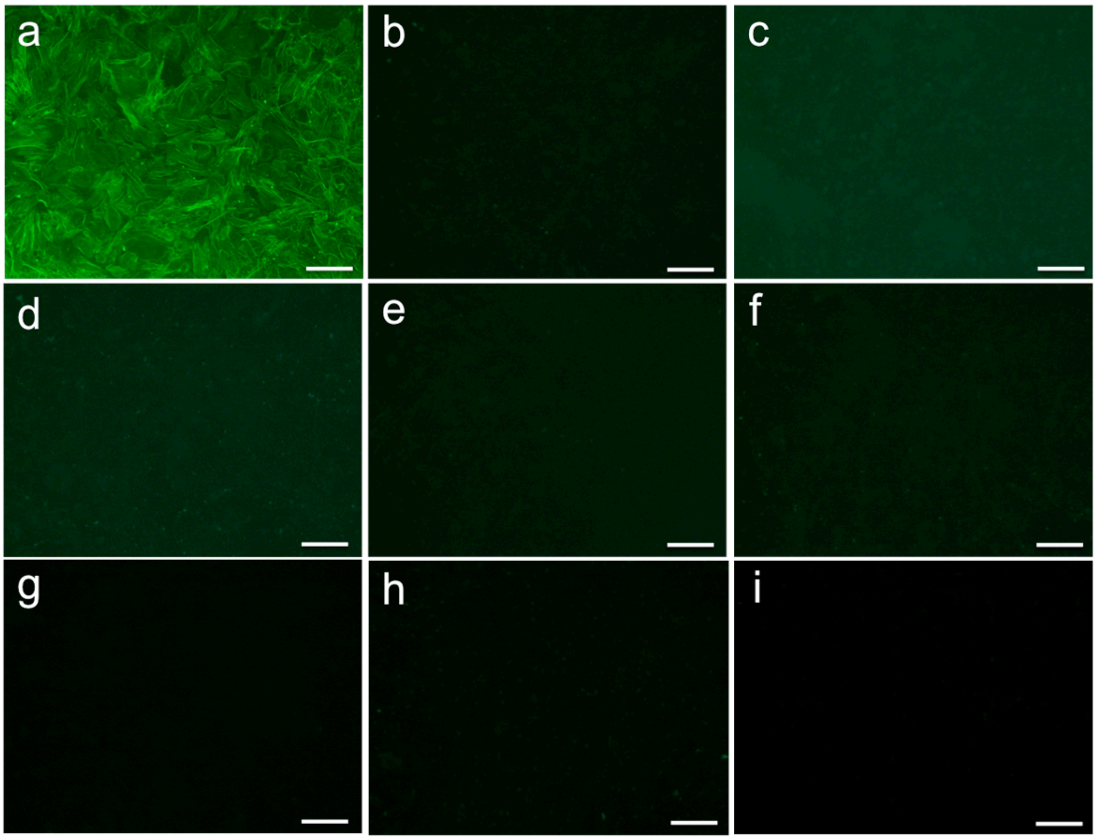

Figure S1. Negative controls of the antibodies used for immunohistochemistry.

Mouse embryonic fibroblasts (MEFs) used for immunohistochemistry. (a) MEFs, are clearly stained for β-actin, as a positive control. MEFs stain negative with antibodies against BRN3B, ATOH7, ISLET1, SNCG, TUJ1, TAU, NFL, and GAP43 ((b), (c), (d), (e), (f), (g), (h), and (i), respectively). Scale bars, 200 μm.

Figure S2

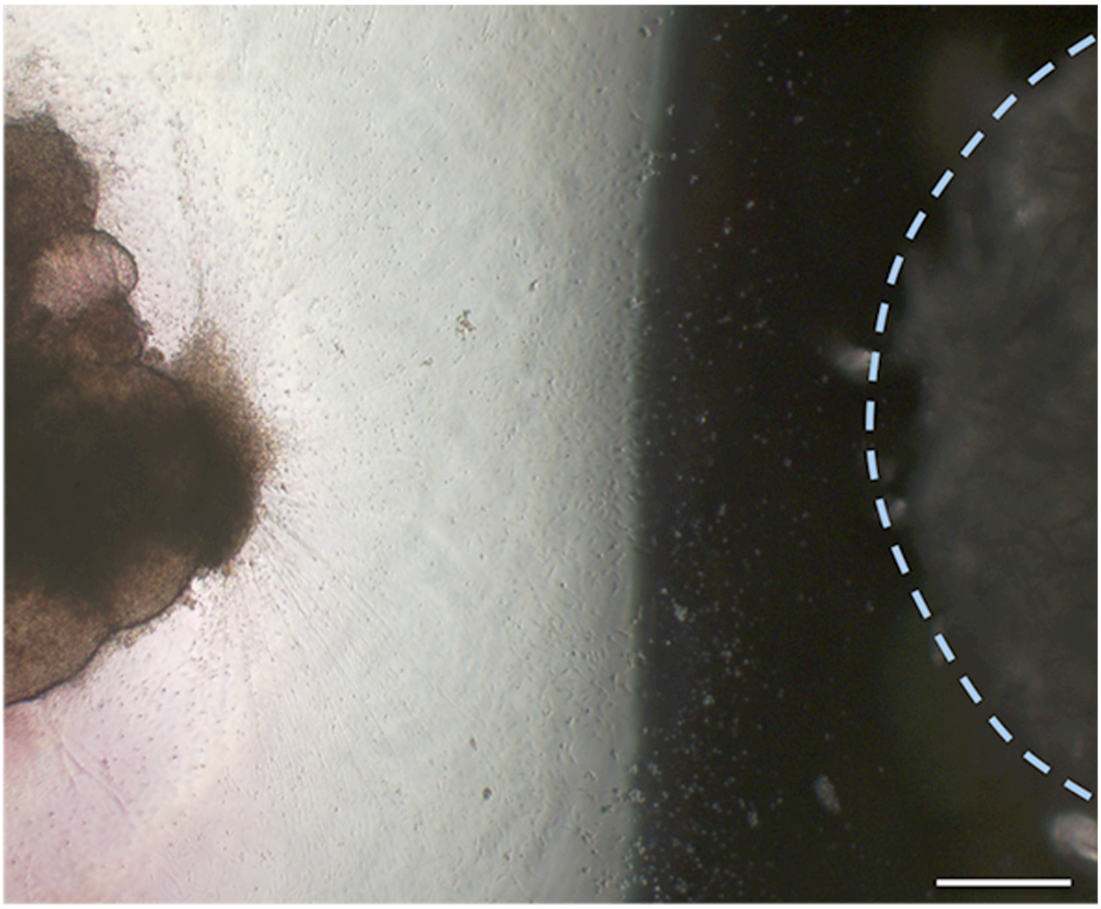

Figure S2. The location of the hydrogel for locally sustained release of neuroactive agents.

The embryoid body attaches to the center of the 24-well dish on D27. On D29, hydrogel containing neuroactive agents is placed at the corner of each well and immediately in front of the elongated axons that develop from the attached optic vesicle. Scale bars, 400 μm.

Figure S3

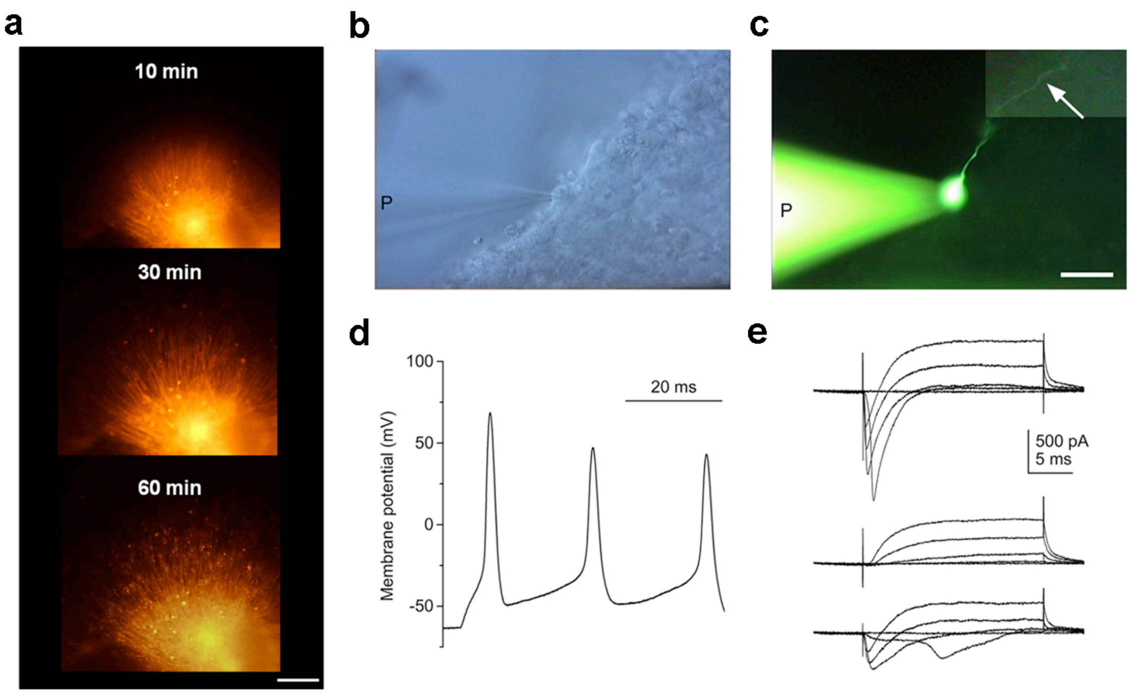

Figure S3. Axonal transport and action potentials of RGCs.

(**a**) Axonal transport in hESC-derived RGCs. Anterograde transport is evaluated by Alexa-Fluo-555-conjugated cholera toxin B injection into the central portion of the attached OVs. Anterograde transport is observed at 10 min and cholera toxin B has spread to the peripheral axons at 60 min. (**b**), (**c**), (**d**), and (**e**) Whole-cell patch recordings and action potentials of RGCs. (**b**) Slice preparation of a hESC-derived RGC colony with a recording electrode, visualised by DIC optics. (**c**) Composite photograph of hESC-derived RGCs with long axon-like processes (arrow) filled with lucifer yellow (LY) under fluorescence. The same field is shown in (**b**) and (**c**). Scale bar is 30 μm. P

indicates the patch pipette. (**d**) Whole-cell patch recording from hESC-derived RGCs reveals action potentials in the current-clamp mode (current injection of 70 pA). (**e**) A family of currents is recorded in the voltage-clamp mode in response to step depolarisation increasing from −51 to +9 mV in 20-mV increments at a holding potential of −71 mV (upper traces). Fast inward currents are blocked by 1 μM TTX (middle traces) and partially recovered after wash out (lower traces). Action potentials and inward currents are recorded from the LY-labelled cell shown in (**c**).

Figure S4

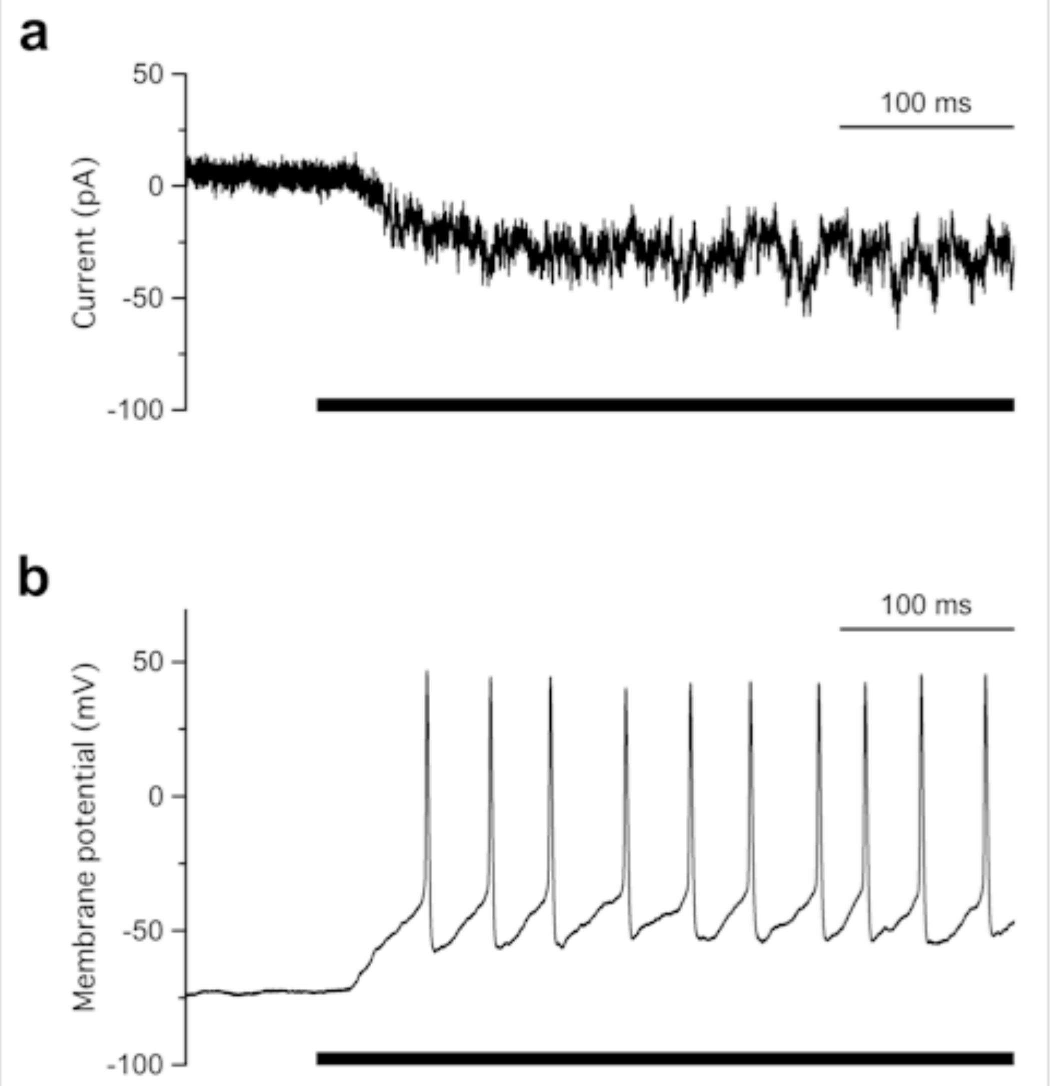

ß

Figure S4. The effect of glutamate on human EC-derived RGCs.

(**a**) When a RGC was voltage-clamped at a holding potential of -63 mV, which is equivalent to the chloride equilibrium potential, an inward current was activated in response to application of glutamate. (**b**) In current-clamp mode, at a membrane potential of ~-75 mV, the puff application depolarized the RGC and induced repetitive action potentials. Black bars in (**a**) and (**b**) indicate the puff application.

Figure S5

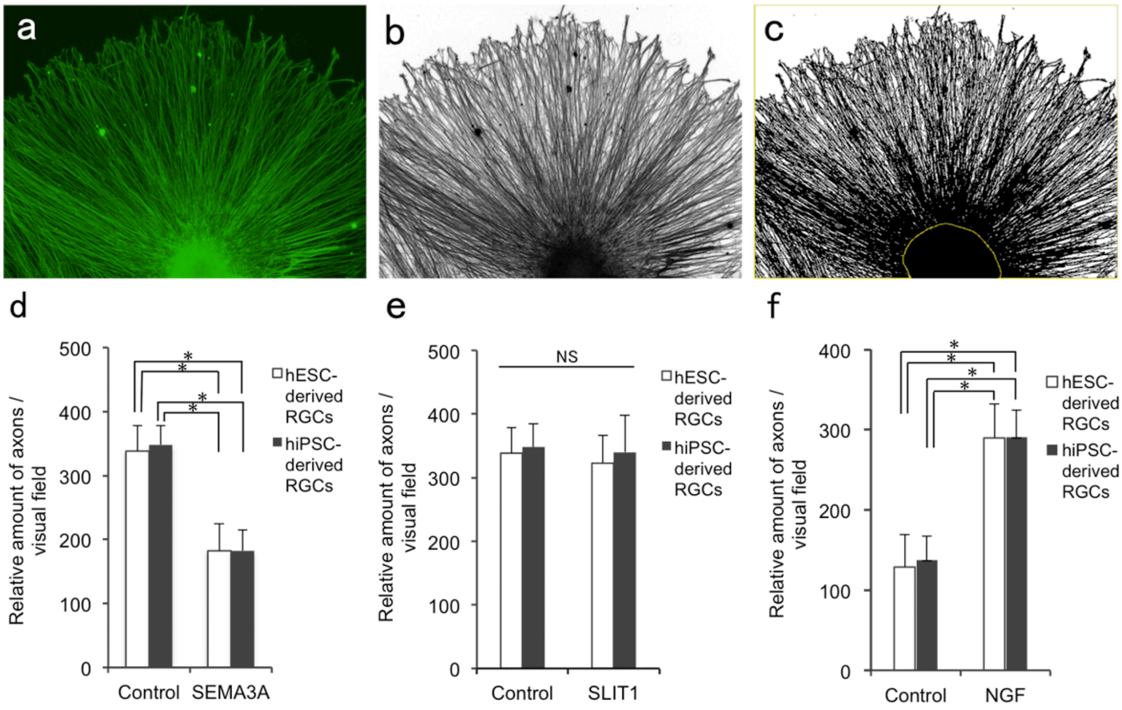

Figure S5. Quantification of the effect of the agents' supplementation on axonal growth of hESC- and hiPSC-derived RGCs.

In order to quantify the amount of axons, we use the algorism indicated from (a) to (c). (a) Firstly, immunohistochemistry for NFL is performed on elongated axons on D30. (b) The images of immunohistochemistry are converted to gray scale images. (c) Finally, the gray-scale images are transformed to binarized images using Image J software (Image J Fiji). The relative number of axons except for the attached OV per visual field, the closed area indicated by the yellow line, is measured. (d) Supplementation with 200 ng/ml SEMA3A from D27 to D30 significantly decreased the number of elongated

axons (ANOVA, $p \leq 0.05$), which did not differ between hESC-derived RGCs and hiPSCs-derived RGCs. (e) Supplementation with 5 μg/ml SLIT1 from D27 to D30 does not affect the number of axons, which did not differ between hESC-derived RGCs and hiPSCs-derived RGCs. (f) Supplementation with 200 ng/ml NGF from D24 to D30 significantly increased the number of elongated axons (ANOVA, $p \leq 0.05$), which did not differ between hESC-derived RGCs and hiPSCs-derived RGCs. Supplementation with NGF was initiated on D24 without FBS or retinoic acid. On D27, attachment of floating EB was achieved without FBS or BDNF, which was used in the SEMA3A and SLIT1 assay. Error bars indicate ± standard deviation (SD). Each column shows an average value for the studied samples ($n = 5$). Scale bars, 200 μm.

Figure S6

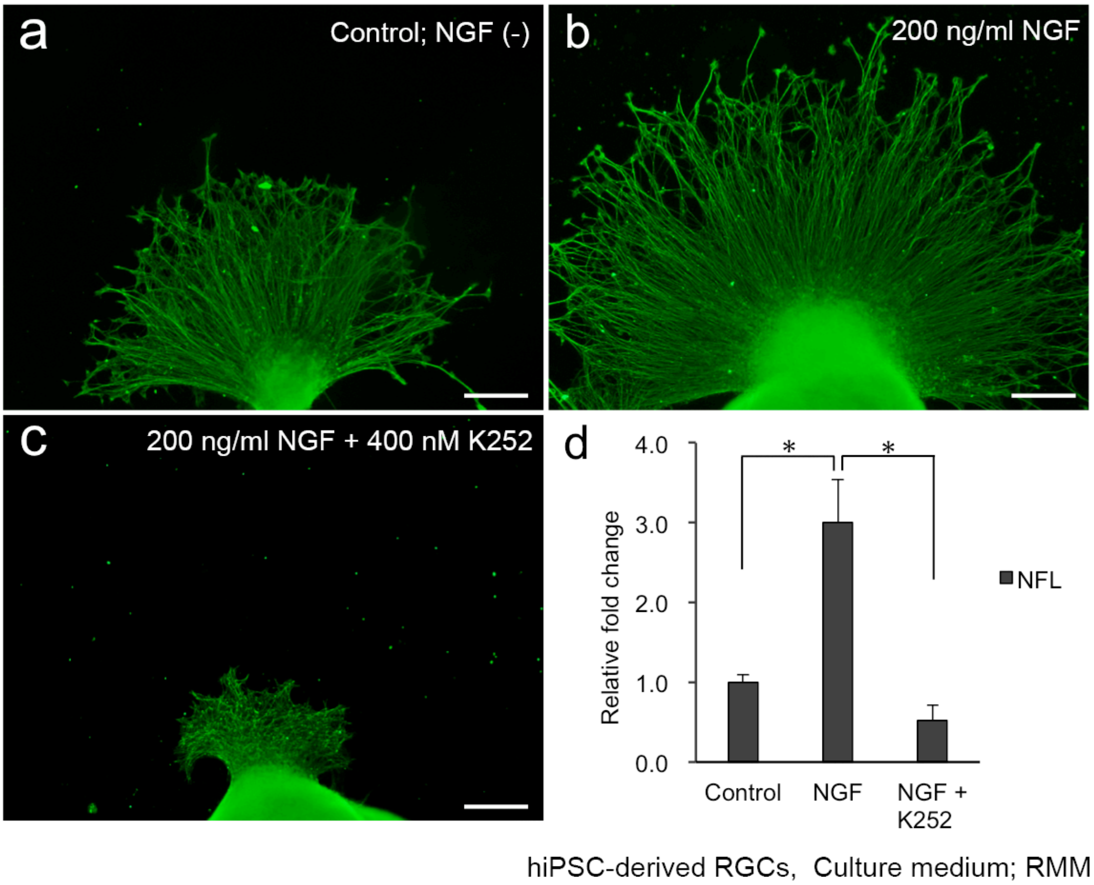

Figure S6. Effect of NGF and K252 supplementation on axonal growth of hESC-derived RGCs

The effect of NGF and its inhibitor, K252, on axonal growth of hESC- derived RGCs was investigated. For this assay, supplementation with NGF was initiated on D24 without FBS or retinoic acid. On D27, attachment of floating EBs was achieved without FBS or BDNF. (a) Control axonal growth of hESC- derived RGCs without NGF supplementation, only with RMM culture medium, is shown. (b) Axonal growth of hESC-derived RGCs with 200 ng/ml NGF supplementation from D24–30 is promoted

compared to the control. (c) Axonal growth of hESC-derived RGCs with 200 ng/ml NGF is clearly inhibited by 400 nM K252 supplementation. (**d**) Quantitative analysis of mRNA expression of *NFL*. Expression of *NFL* is significantly upregulated by NGF supplementation, and the expression is decrease to the control level by 400 nM K252 (ANOVA, $p \leq 0.05$). Error bars indicate ± standard deviation (SD). Each column shows an average value for the studied samples (n = 5). Scale bar, 200 μm.

Figure S7

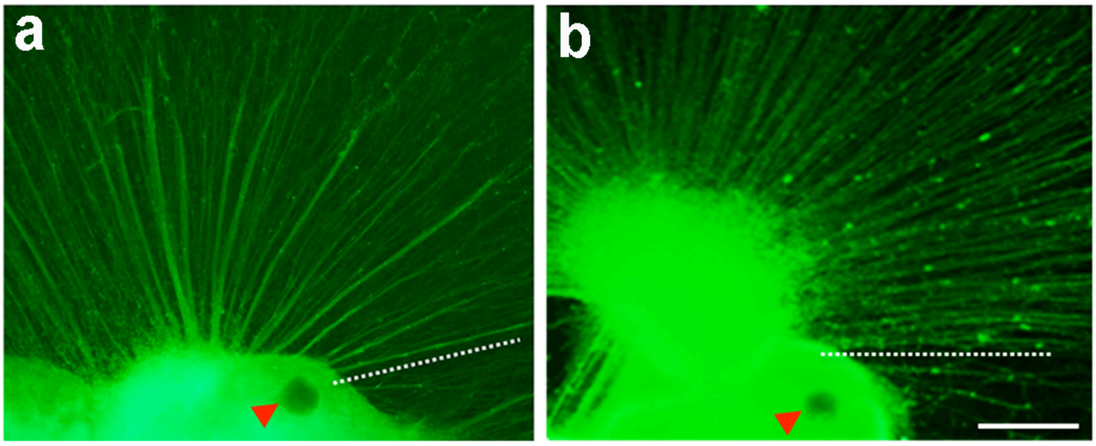

Bead; 200 ng/ml NGF
hESC-derived RGCs

Bead; 200 ng/ml NGF
hiPSC-derived RGCs

Figure S7. The effect of focally sustained release of NGF on pathfinding of axons of hESC- and hiPSC-derived RGCs.

Immunohistochemistry of NFL reveals that the axonal path of hESC- and hiPSC-derived RGCs located nearest to the beads, indicated by dashed lines, ((**a**) and (**b**), respectively), are not influenced by focal NGF release from transplanted beads. Beads are not stained by NFL and form the round and black area indicated by red arrowheads in (**a**) and (**b**). Scale bar, 100 µm.

Figure S8

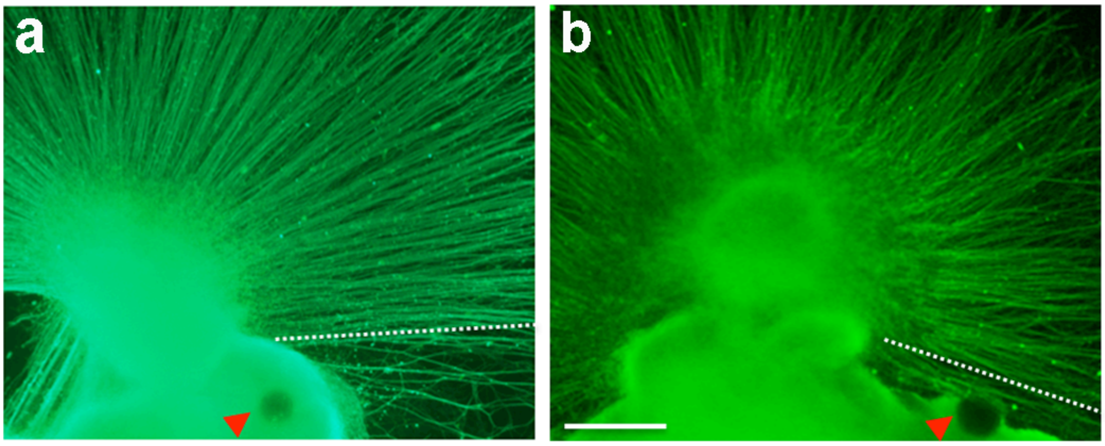

Bead; 200 ng/ml SEMA3A
hESC-derived RGCs

Bead; 200 ng/ml SEMA3A
hiPSC-derived RGCs

Figure S8. The effect of focally sustained release of SEMA3A on pathfinding of axons of hESC- and hiPSC-derived RGCs.

Immunohistochemistry of NFL reveals that the axonal path of hESC- and hiPSC-derived RGCs located nearest portion from the beads, indicated by dashed lines ((**a**) and (**b**), respectively), are not repelled by focal SEMA3A release. Beads are not stained by NFL and form the round and black area indicated by red arrowheads in (**a**) and (**b**). Scale bar, 100 μm.

Figure S9

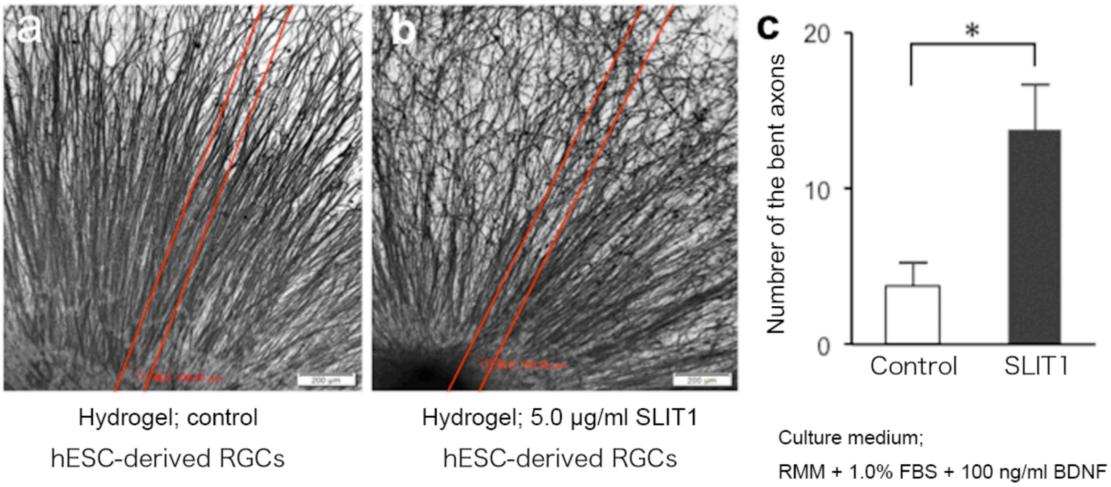

Figure S9. Quantification of the bent axons by local sustained release of SLIT1 (5 μg/ml) from hydrogels placed in front of the attached OVs.

(a)Radiated axons of hESC-derived RGCs on D31, stained for NFL, are converted in gray scale. In the control, few axons take a path through the 100-μm-wide area, parallel to the irradiated axons (red lines). (b) Compared to the control, bent axons are clearly identified and take a path through the 100-μm-wide area by the local sustained release of 5 μg/ml SLIT1, placed immediately in front of the irradiated axons. (c) The number of bent axons that take a path through the 100-μm-wide area is significantly increased by local SLIT1 release (*t*-test, $p \leq 0.05$). Error bars indicate ± standard deviation (SD). Each column shows an average value for the studied samples (n = 4). Scale bars, 200 μm.

Supplementary Table

Table S1. Primer list. The primer sets are used for real-time PCR analysis.

	Gene	Accession No	Forward (5′ –3′)	Reverse (5′ –3′)
1	*HPRT1*	NM_000194.2	GGCAGTATAATCCAAAGATGGTCAA	GTCAAGGGCATATCCTACAACAAAC
2	*BRN3B*	NM_004575.2	TGACACATGAGCGCTCTCACTTAC	ACCAAGTGGCAAATGCACCTA
3	*ATHO7*	NM_145178.3	CCCTAAATTTGGGCAAGTGAAGA	CAAAGCAACTCACGTGCAATC
4	*TUJ1*	NM_006086.3	GGCCAAGGGTCACTACACG	GCAGTCGCAGTTTTCACACTC
5	*ISLET1*	NM_002202.2	GCGGAGTGTAATCAGTATTTGGA	GCATTTGATCCCGTACAACCT
6	*SNCG*	NM_003087.2	TGAGCAGCGTCAACACTGTG	GAGGTGACCGCGATGTTCTC
7	*NFL*	NM_006158.4	TCAACGTGAAGATGGCTTTGGATA	AAGACCTGGGAGCTCTGGGAGTA
8	*TAU*	NM_010838.4	ACCCCATCCCTACCAACAC	CAGGCGGCTCTTACTAGCTG

Supplementary Videos

Video S1. Time-lapse analysis of axonal transport in RGCs derived from hESCs.

A time-series of axonal transport is also recorded by the injection of Alexa Fluor 555-conjugated cholera toxin B into the central portion of the clump of cells. Fast and slow flows are identified.

Video S2. Time-lapse analysis of axonal pathfinding of hiPSC-derived RGCs repelled by SLIT1.

Axonal path of hiPSC-derived RGCs is viewed over 18 h. Initially, one axon progresses straight towards the SLIT1-releasing hydrogel but retracts after 9 h, and disappears after 18 h. Another axon progresses straight towards the SLIT1-releasing hydrogel after 9 h, but redirects its path to the left after 18 h.

Supplementary Reference

1. Tanaka, T. *et al.* Generation of retinal ganglion cells with functional axons from human induced pluripotent stem cells. *Sci Rep* **5**, 8344 (2015).
